# *Isocitrate dehydrogenase 1* mutations *(IDH1)* and *p16/CDKN2A* copy number change in conventional chondrosarcomas

**DOI:** 10.1007/s00428-014-1685-4

**Published:** 2014-11-29

**Authors:** M. Fernanda Amary, Hongtao Ye, Georgina Forbes, Stephen Damato, Francesca Maggiani, Robin Pollock, Roberto Tirabosco, Adrienne M. Flanagan

**Affiliations:** 1 Department of Histopathology and Surgery, Royal National Orthopaedic Hospital, NHS Trust, Brockley Hill, HA7 4LP Stanmore, Middlesex UK; 2 Cancer Institute, University College London, Huntley Street, WC1E 6BT London, UK; 3 Paul O’Gorman UCL Cancer Institute, University College London, Huntley Street, WC1E 6BT London, UK

**Keywords:** Chondrosarcomas, IDH1, *p16/CDKN2A*, Sarcoma

## Abstract

To determine whether *IDH1* mutations are present in primary and relapsed (local and distal) conventional central chondrosarcomas; and secondly, to assess if loss of *p16/CDKN2A* is associated with tumour grade progression, 102 tumour samples from 37 patients, including material from presenting and relapse events, were assessed. All wild-type cases for *IDH1* R132 substitutions were also tested for *IDH2* R172 and R140 alterations. The primary tumour and the most recent relapse sample were tested for *p16/CDKN2A* by interphase fluorescence in situ hybridisation. An additional 120 central cartilaginous tumours from different patients were also tested for *p16/CDKN2A* copy number. The study shows that if an *IDH1* mutation were detected in a primary central chondrosarcoma, it is always detected at the time of presentation, and the same mutation is detected in local recurrences and metastatic events. We show that *p16/CDKN2A* copy number variation occurs subsequent to the *IDH1* mutation, and confirm that *p16/CDKN2A* copy number variation occurs in 75 % of high grade central chondrosarcomas, and not in low grade cartilaginous tumours. Finally, *p16/CDKN2A* copy number variation is seen in both the *IDH1* wild-type and mutant cartilaginous central tumours.

## Introduction

Isocitrate dehydrogenase 1 (*IDH1*), and *IDH2* mutations were originally reported as frequent events in gliomas and acute myeloid leukaemia. We subsequently revealed the presence of the same mutations in at least 56 % of central chondrosarcomas: the mutations are present in all grades and the dedifferentiated variant but not in the other subtypes (peripheral and clear cell chondrosarcoma), or in soft tissue tumours [1, 6]. The mutations are also present in enchondromas, the benign central cartilaginous tumour considered to represent the precursor of central chondrosarcoma, and occur as early post-zygotic events in the common forms of multiple enchondromas, namely Ollier disease and Maffucci syndrome [3, 9]. These findings make a case for *IDH1/2* mutations occurring early in the development of the non-syndromic disease and that the mutations represent driver mutations, to which the tumours are addicted for survival. Nevertheless, to provide further evidence for this, we now evaluate if the mutations always occur in the presenting tumour, and if they persist in recurrent and metastatic disease. This is an important question if drugs, which may be developed against the mutant protein, are to be effective [10].

Disease progression in *IDH1/2* mutant-bearing tumours is likely to depend on additional contributing genetic events. Loss of copy number of *p16/CDKN2A* is a well recognised tumour suppressor gene involved in cancer oncogenic process, and has been reported to be associated with disease progression of cartilaginous tumours. Specifically, *p16/CDKN2A* has been found to occur in high grade chondrosarcomas but rarely in chondrosarcoma grade (G) I and not in enchondromas [5, 13]. Furthermore, we subsequently revealed that homozygous *p16/CDKN2A* deletions were only detected in high grade chondrosarcomas (GII, GIII and dedifferentiated) but not in GI central tumours, and that these genetics alterations were detected in both *IDH1* mutant and wild-type (WT) central chondrosarcomas [12]. Therefore, we wished to determine if loss of *p16/CDKN2A* in relapse disease correlates with progression to a higher grade of tumour.

## Material and methods

One hundred and two samples from 37 patients, with the associated demographic data, and the interval between the primary tumour and first relapse event, were retrieved from the files of the Royal National Orthopaedic Hospital NHS Trust. Haematoxylin and eosin-stained sections from all selected tumours were reviewed (MFA, AMF, RT). Age and gender of the 37 patients with their tumour grade are shown in Table 1.

**Table 1:** Characteristics of 37 patients with multiple samples

Case	Age	Gender	Site primary	Number of samples	Grade primary	Grade relapses	*IDH1* status	CDKN2A/p16	Interval from primary to 1st relapse (months)
1	32	female	sacrum	3	G2	G2	WT	loss > further loss	31
2	80	male	femur	4	Dediff	Dediff	R132H	normal > loss	24
3	35	male	pelvis	2	G2	G2	WT	loss	51
4	50	male	vertebra	3	G2	G2	WT	NI	102
5	69	female	rib	4	G2/3	G2/3	WT	NI	15
6	41	female	sternum	2	G2	G3	WT	normal	101
7	39	female	tibia	2	G2	G2	WT	normal	88
8	57	male	rib	4	G2	G2	R132C	normal	8
9	30	male	scapula	2	G2	Dediff	WT	loss > polysomic	17
10	41	male	vertebra	3	G3	G3	WT	loss	27
11	44	male	pelvis	3	G3	G3	R132G	polysomic	19
12	22	male	sacrum	3	G2	G2	WT	loss	12
13	51	male	humerus	3	G3	G3	WT	normal > polysomic	9
14	74	male	femur	2	G2	G2	R132G	loss	38
15	42	female	hand	2	G2	G2	R132S	NI	67
16	39	male	humerus	3	G2	G3	R132C	normal > loss	15
17	43	male	femur	2	G2	G2	R132C	normal	57
18	64	female	tibia	3	G1	G1	WT	NI	14
19	55	male	hand	6	G2	G3	R132G	normal	15
20	28	female	pelvis	2	G2	G2	WT	loss	34
21	31	male	scapula	5	G2	G3	WT	normal > loss	15
22	69	female	femur	3	G2	G3	R132G	NI	13
23	43	male	vertebra	4	G2	G2	WT	normal	11
24	34	male	humerus	2	G2	G3	WT	normal > loss	16
25	50	male	humerus	3	G3	G3	WT	loss	12
26	77	male	tibia	2	G3	G3	R132L	loss	33
27	72	male	femur	2	Dediff	Dediff	R132H	loss	6
28	58	female	sacrum	2	G2	G2	WT	normal	27
29	36	female	humerus	2	G1	G1	R132C	normal	15
30	51	male	femur	3	Dediff	Dediff	R132H	loss	5
31	52	male	pelvis	2	Dediff	Dediff	R132C	polysomic	12
32	55	male	femur	2	Dediff	Dediff	R132G	loss	4
33	30	female	tibia	3	G2	G2	WT	normal	48
34	68	male	femur	2	Dediff	Dediff	R132S	polysomic	1
35	61	male	femur	2	G2	G2	R132H	normal > loss	23
36	65	male	rib	2	G2	Dediff	WT	NI	26
37	29	female	femur	3	G2	G2	R132C	normal	127

*G* grade, *Dediff* dedifferentiated chondrosarcomas, *WT* wild-type

An additional 120 central conventional cartilaginous tumours from our previous *IDH* study were analysed for *p16/CDKN2A* copy number [1]. This cohort included 61 low grade cartilaginous tumours (enchondromas and chondrosarcomas GI), 2 chondrosarcomas GI with transition to GII, 30 chondrosarcomas GII, 6 chondrosarcomas GIII and 21 dedifferentiated chondrosarcomas.

Genomic DNA was extracted from material that was at least 60 % tumour-rich and was analysed using PCR amplification followed by capillary sequencing and/or realtime PCR as previously described [1]. Sections from the primary tumour and the most recent recurrence were analysed by fluorescence in situ hybridisation (FISH) for *p16/CDKN2A* (9p21) using commercially available probes (Vysis, Abbott Molecular, Illinois USA) as previously described [2]. Loss of *p16/CDKN2A* was defined as: (i) monosomy (1 *p16/CDKN2A* and 1 centromeric signal); (ii) heterozygous deletion (loss of 1 copy of *p16/CDKN2A* in the presence of 2 centromeric signals) and (iii) homozygous deletion (loss of 2 copies of *p16/CDKN2A* in the presence of 1 or 2 centromeric signals) in at least 15 % of the nuclei analysed (minimum count of 50 consecutive non-overlapping nuclei). Polysomy was defined as more than 2 copies of *p16/CDKN2A*/CEP 9 in at least 15 % of the nuclei analysed.

## Results

**Fig. 1:**
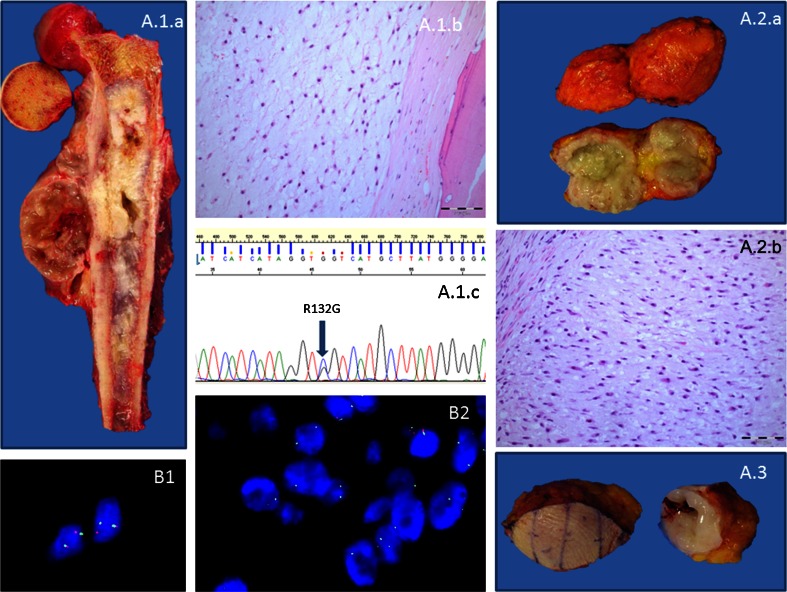
**a** Case 22 showing the primary (*A.1.a*) excision of a chondrosarcoma GII, (*A.1.b*) harbouring a R132G substitution (*A.1.c*) and 2 subsequent relapse events in the chest wall (*A.2.a*, *A.2.b* and *A.3*). **b** Case 30. Diploid *p16/CDKN2A* copy number (*B1*) and heterozygous loss of *p16/CDKN2A* (*B2*)

One hundred and two tumour samples from 37 patients were analysed for *IDH1* R132 of which 48 (49 %) were found to harbour a R132 *IDH1* mutation. Thirty of these 48 tumours represented disease from relapse events (27 local recurrences and 3 metastatic events). An *IDH1* mutation was detected in the presenting and relapse samples from each of these 18 patients. All samples from a given patient in this group had the same *IDH1* alteration (Fig. 1a.). If a tumour at presentation was found to be wild-type (WT) for *IDH1* R132 mutations, the relapse samples were also WT (19/37 patients). In *IDH1* WT cases, at least 1 sample, from each of these patients, was also screened for both *IDH2* R172 and R140 mutations. No *IDH2* (R172 or R140) mutations were detected in this cohort.

Details of the interval between the primary treatment (curettage or wide excision) of the presenting tumour and the first recurrence is given in Table 1. The number of relapse events per patient ranged from 1 to 5 (average 1.9).

Analysis of the *p16/CDKN2A* gene locus by FISH gave informative results from both the primary tumour and the most recent relapse in 31 of the 37 patients. In contrast to the persistent presence of *IDH1* mutations throughout tumour progression, *p16/CDKN2A* copy number variation only occurred in high grade chondrosarcomas (GII, GIII or dedifferentiated neoplasms). Of the 21 tumour sets (from 31 informative cases) in which *p16/CDKN2A* copy number variation was identified in either the primary and/or relapse event, 11 harboured an *IDH1* mutation and 10 were WT for *IDH1* and *IDH2*, indicating that change in *p16/CDKN2A* copy number is equally common in *IDH1* mutant and WT central conventional cartilaginous tumours.

Of the 31 informative tumour sets, 23 revealed the same *p16/CDKN2A* copy number in both samples, and 8 had discordant copy number (*vide infra*). The former included 10 with normal copy number of *p16/CDKN2A*, 10 with loss of *p16/CDKN2A*, and 3 with polysomy for chromosome 9. In only 2 of these tumour sets did the grade of the tumour progress; in both cases from GII to GIII, both with normal copy number of *p16/CDKN2A* (Table 1). There were only 2 chondrosarcoma GI which recurred, 1 was non-informative for FISH, and the other revealed a normal copy number of *p16/CDKN2A*. Within this group, the samples from patient 30 were particularly noteworthy in that, the presenting tumour was a dedifferentiated chondrosarcoma in which the normal (diploid) *p16/CDKN2A* copy number was detected in the well-differentiated component (Fig. 1. b1; Table 1) and a homozygous deletion was detected in both the dedifferentiated component of the presenting and the metastatic tumour (Fig. 1. b2).

Of the 8 sample sets from the 31 patients which revealed discordant *p16/CDKN2A* copy number, loss of *p16/CDKN2A* was observed in the relapse event and not in the primary tumour in 5 cases (3 of which also harboured *IDH1* mutation). In tumours from 2 patients, polyploidy of chromosome 9 was observed in the relapse event whereas in the primary tumour, the normal copy number was seen in 1 case and monosomy in the other. In the remaining case, loss of *p16/CDKN2A* converted from being heterozygous in the primary tumour to being homozygous in the relapse sample. In 4/8 (50 %) of these patients, progression of the chondrosarcoma grade occurred in the relapse. In 3, the primary tumour showed normal copy number, and progression to a higher morphological grade (GII to GIII) was accompanied by *p16/CDKN2A* loss. The fourth tumour which presented as a chondrosarcoma GII was monosomic for chromosome 9 and progressed to a dedifferentiated tumour, in which the tumour cells were polysomic for chromosome 9.

The additional 120 central conventional cartilaginous tumours from 120 different patients were analysed for *p16/CDKN2A* by FISH. Of the 61 low grade tumours, all revealed normal diploid *p16/CDKN2A* copy number. Loss of *p16/CDKN2A* was detected in 25 % (8/30) of the CHS GII, including 2 cases diagnosed as GI with transition to GII, and 50 % of the CHS GIII (3/6). The 62 % of the dedifferentiated chondrosarcomas (13/21) also revealed *p16/CDKN2A* loss. Polysomy of *p16/CDKN2A* was detected in 2/30 GII, 1/6 GIII and 6/21 dedifferentiated chondrosarcomas.

Further analysis of 3/21 dedifferentiated chondrosarcomas where material was available/suitable for analysis, there was normal diploid *p16/CDKN2A* copy number in the well-differentiated component and *p16/CDKN2A* loss in the dedifferentiated component.

## Discussion

In this study, we confirm the data published in our previous report showing that *IDH1* mutations occur in ∼50 % of solitary conventional central chondrosarcomas, that the *IDH1* mutations are found in all grades of this subtype of chondrosarcoma, that the R132C mutation is the most common genetic alteration in this gene, that *IDH2* mutations are significantly less common than *IDH1* mutations and that to date an *IDH2* R140 mutation, which is seen in acute myeloid leukaemia, is not found in cartilaginous tumours [1]. In addition, we now show that when an *IDH1* mutation is present in a solitary primary central conventional chondrosarcoma, the same mutation is always detected in local recurrences and metastatic events. Furthermore, a mutation is never detected in a locally recurrent tumour or metastatic lesion when it is not detected in the presenting tumour. These data add weight to existing evidence that the R132 *IDH1* mutations occur early in the genesis of cartilaginous tumours and are therefore important driver alterations. This provides a strong argument that the cartilaginous tumours harbouring the genetically altered *IDH1* are good candidates for the selective inhibitors of the metabolic enzyme, IDH1 [10].

Although *IDH1/IDH2* mutations appear to be an important genetic event in the pathogenesis of enchondromas and central chondrosarcomas, other mutations are likely to exert an impact on the biological behaviour of the disease. To date, two important tumour suppressor genes, *p16/CDKN2A and TP53* have been implicated in the development of high grade chondrosarcoma [5, 11, 12]. Genetic alterations of both of these genes have been detected previously in chondrosarcomas [4, 5, 12]. In the case of *TP53*, inactivation has been shown to occur as a result of chromosomal loss and point mutations, and *TP53* mutations have been detected in both *IDH1* WT and mutant chondrosarcomas [8, 12]. Herein, we demonstrate that alteration in p16/CDKN2A copy number occurs exclusively in high grade central cartilaginous tumours, and that the percentage of cases with alteration of copy number of *p16/CDKN2A* increases with grade. Specifically, we show that 75 % (67 tumours from 89 patients) of central high grade chondrosarcomas reveal loss or gain (polysomy) of *p16/CDKN2A* in at least one sample (data generated from both cohorts). We also show that loss of *p16/CDKN2A* is found in both *IDH1* WT and mutant cartilaginous central tumours, indicating that the two genetic events are unrelated.

We consider that our findings support the concept that alteration of *p16/CDKN2A* copy number occurs subsequent to the acquisition of the *IDH1* mutation. This is supported by our findings that in 60 low grade central conventional tumours (enchondroma/low grade chondrosarcoma), obtained from patients with a single tumour sample, none had *p16/CDKN2A* copy number variation. This concurs with previously published data from smaller cohorts which report that loss of *p16/CDKN2A* is rarely present in enchondromas and low grade chondrosarcomas, whereas *IDH1* and *IDH2* mutations are found in tumours of all grades of chondrosarcomas and in benign conventional central cartilaginous tumours [12, 13]. Our findings indicate that if a tumour shows *p16/CDKN2A* copy number variation (gain or loss), it should be managed as a high grade chondrosarcoma. As grading of chondrosarcoma has been shown to lack reproducibility [7] assessment of alteration in copy number of *p16/CDKN2A* can be used to complement histological grading of chondrosarcoma. The presence of alteration in copy number argues strongly for a diagnosis of high grade disease, although the absence of such abnormality is not helpful as such changes are detected in ∼75 % of high grade chondrosarcoma. Future studies would be helpful to identify a biomarker to distinguish the remaining 25 % of high grade chondrosarcomas from low grade disease.

We conclude that *IDH1* mutations represent a recurrent genetic event in solitary central chondrosarcomas, that they occur early in disease development and persist during tumour recurrence and metastatic disease thereby adding to the existing evidence that such mutations represent drivers of disease. The *p16/CDKN2A* copy number variation occurs in at least 50 % of *IDH1* mutant-bearing high grade chondrosarcomas, and the findings support the concept that the *p16/CDKN2A* alterations are likely to be associated with disease progression. Assessment of *p16/CDKN2A* copy number can be used as an ancillary test to help reach a conclusion about the grade of a chondrosarcoma thereby providing additional information on which to decide clinical management of this disease.
